# Supplementary material for: P-TRAP: a Panicle Trait Phenotyping tool
Source: BMC Plant Biol. 2013 Aug 29;13:122. doi: 10.1186/1471-2229-13-122 (PMC3848748; doi:10.1186/1471-2229-13-122)
Supplement: Additional file 7 — P-TRAP source code. The Netbeans project that contains the source code of the application. [file 1471-2229-13-122-S7.zip › javadoc.zip!/javadoc/org-bioalg-graphprocessor/index-all.html]

Index (GraphProcessor)


JavaScript is disabled on your browser.


- Package
- Class
- Tree
- Deprecated
- Index
- Help

*org.bioalg.graphprocessor 1.0*

- Prev
- Next

- Frames
- No Frames

- All Classes

A B C E F G H I M O P R S T V 


## A

add(Vertex) - Method in class org.bioalg.graphprocessor.Path


add(int, Vertex) - Method in class org.bioalg.graphprocessor.Path


addEdge(Edge) - Method in class org.bioalg.graphprocessor.Graph


addEdge(Vertex, Vertex) - Method in class org.bioalg.graphprocessor.GraphModel


addvetex(Vertex) - Method in class org.bioalg.graphprocessor.GraphModel


## B

BranchModel - Class in org.bioalg.graphprocessor
:   A general class for representing branches.

BranchModel(Vertex, Vertex) - Constructor for class org.bioalg.graphprocessor.BranchModel


BranchModel(Vertex, Vertex, GraphModel) - Constructor for class org.bioalg.graphprocessor.BranchModel


BranchModel(Vertex) - Constructor for class org.bioalg.graphprocessor.BranchModel


BranchModel() - Constructor for class org.bioalg.graphprocessor.BranchModel


## C

calclastGenereatingPoint() - Method in class org.bioalg.graphprocessor.Graph
:   Calculate the generating last generating point in the graph.

calcpath() - Method in class org.bioalg.graphprocessor.BranchModel
:   calculates path of the branch in the graph

calcStartandEndVertices(Vertex) - Method in class org.bioalg.graphprocessor.BranchModel
:   calculate the start and end vertices, .

clear() - Method in class org.bioalg.graphprocessor.Graph


clear() - Method in class org.bioalg.graphprocessor.Path


clearall() - Method in class org.bioalg.graphprocessor.GraphModel


compareTo(Object) - Method in class org.bioalg.graphprocessor.Edge


contains(Vertex) - Method in class org.bioalg.graphprocessor.Path


## E

Edge - Class in org.bioalg.graphprocessor
:   A general edge class.

Edge(Point, Point) - Constructor for class org.bioalg.graphprocessor.Edge


Edge.EDGE\_TYPE - Enum in org.bioalg.graphprocessor


EdgeModel - Class in org.bioalg.graphprocessor
:   Similar to the `Edge` class but is using vertices instead of points.

EdgeModel(Vertex, Vertex) - Constructor for class org.bioalg.graphprocessor.EdgeModel


equals(Object) - Method in class org.bioalg.graphprocessor.Edge


## F

fixPrimaryJunctions() - Method in class org.bioalg.graphprocessor.GraphModel


## G

get(int) - Method in class org.bioalg.graphprocessor.Path


getAllBranches() - Method in class org.bioalg.graphprocessor.GraphModel


getChildren(boolean) - Method in class org.bioalg.graphprocessor.BranchModel
:   Return all sub branches of the branch

getEdge(int) - Method in class org.bioalg.graphprocessor.Graph


getEdges() - Method in class org.bioalg.graphprocessor.Graph


getEdges() - Method in class org.bioalg.graphprocessor.GraphModel


getEndVertex() - Method in class org.bioalg.graphprocessor.BranchModel


getfixedPoints() - Method in class org.bioalg.graphprocessor.GraphModel


getid() - Method in class org.bioalg.graphprocessor.Vertex


getLastGenereatingPoint() - Method in class org.bioalg.graphprocessor.Graph


getLength() - Method in class org.bioalg.graphprocessor.BranchModel


getLength() - Method in class org.bioalg.graphprocessor.Graph


getlength() - Method in class org.bioalg.graphprocessor.Path


getMainbaranches() - Method in class org.bioalg.graphprocessor.Graph


getMainGeneratingPoints() - Method in class org.bioalg.graphprocessor.Graph


getNeighbors(Vertex) - Method in class org.bioalg.graphprocessor.GraphModel


getNeighbors(Vertex, Vertex.VERTEX\_TYPE) - Method in class org.bioalg.graphprocessor.GraphModel


getNeighbors(Vertex, Vertex.VERTEX\_TYPE, Vertex.VERTEX\_TYPE) - Method in class org.bioalg.graphprocessor.GraphModel


getNodes() - Method in class org.bioalg.graphprocessor.Path


getP1() - Method in class org.bioalg.graphprocessor.Edge


getP2() - Method in class org.bioalg.graphprocessor.Edge


getRachisLength(ArrayList<BranchModel>) - Method in class org.bioalg.graphprocessor.GraphModel


getSecondaryBranches(Path) - Method in class org.bioalg.graphprocessor.BranchModel


getStart2endPath() - Method in class org.bioalg.graphprocessor.BranchModel


getStartVertex() - Method in class org.bioalg.graphprocessor.BranchModel


getSubGeneratingPoints() - Method in class org.bioalg.graphprocessor.Graph


getTertiaryBranches(Path) - Method in class org.bioalg.graphprocessor.BranchModel
:   getThe third level branches.

getType() - Method in class org.bioalg.graphprocessor.Edge


getType() - Method in class org.bioalg.graphprocessor.Vertex


getVertex1() - Method in class org.bioalg.graphprocessor.EdgeModel


getVertex2() - Method in class org.bioalg.graphprocessor.EdgeModel


getvertexByid(String) - Method in class org.bioalg.graphprocessor.GraphModel


getVertices() - Method in class org.bioalg.graphprocessor.GraphModel


getVertixNeighbors(Point) - Method in class org.bioalg.graphprocessor.Graph
:   Return the neighbors of a given point

Graph - Class in org.bioalg.graphprocessor
:   The mathematical graph.

Graph() - Constructor for class org.bioalg.graphprocessor.Graph


GraphModel - Class in org.bioalg.graphprocessor
:   A graph model that can be used to warp xml files, this is more portable than the `Graph` class for both visualizing the results and user manual editing.

GraphModel() - Constructor for class org.bioalg.graphprocessor.GraphModel


GraphModel(InputStream) - Constructor for class org.bioalg.graphprocessor.GraphModel
:   Constructor from an `InputStream` object.

GraphModel(Element) - Constructor for class org.bioalg.graphprocessor.GraphModel
:   Constructor from an XML element.

GraphModel.GraphErrors - Enum in org.bioalg.graphprocessor


## H

hasChildlessNode() - Method in class org.bioalg.graphprocessor.GraphModel


hasEdge(Edge) - Method in class org.bioalg.graphprocessor.Graph
:   Return true if the graph contains the input edge.

hasFlyingNodes() - Method in class org.bioalg.graphprocessor.GraphModel


hasTwoFixedNodes() - Method in class org.bioalg.graphprocessor.GraphModel


## I

info - Variable in class org.bioalg.graphprocessor.Edge


isdummy(ArrayList<Vertex>) - Method in class org.bioalg.graphprocessor.GraphModel


isFixed() - Method in class org.bioalg.graphprocessor.Vertex


isGeneratingPoint(Point) - Method in class org.bioalg.graphprocessor.Graph
:   Return true if a given point has more than three neighbors

isTransition(Vertex) - Method in class org.bioalg.graphprocessor.GraphModel


## M

makeDirected() - Method in class org.bioalg.graphprocessor.GraphModel
:   convert it to a directed graph


## O

org.bioalg.graphprocessor - package org.bioalg.graphprocessor


## P

Path - Class in org.bioalg.graphprocessor
:   A path is the list of vertices starts and ends by two vertices.

Path() - Constructor for class org.bioalg.graphprocessor.Path


Path(ArrayList<Vertex>) - Constructor for class org.bioalg.graphprocessor.Path


## R

refineModel(int) - Method in class org.bioalg.graphprocessor.GraphModel
:   Remove crowded nodes at the junctions

removeDuplicates(int) - Method in class org.bioalg.graphprocessor.Graph
:   Remove duplicated vertices if any

removeEdge(Edge) - Method in class org.bioalg.graphprocessor.Graph


removeEdge(EdgeModel) - Method in class org.bioalg.graphprocessor.GraphModel


removeSpikes(int) - Method in class org.bioalg.graphprocessor.Graph
:   Remove the spikes given a length threshold

removetranstions() - Method in class org.bioalg.graphprocessor.Graph
:   Remove the transition vertices from the graph.

removeVertex(Vertex) - Method in class org.bioalg.graphprocessor.GraphModel


## S

setEdges(ArrayList<Edge>) - Method in class org.bioalg.graphprocessor.Graph


setEdges(ArrayList<EdgeModel>) - Method in class org.bioalg.graphprocessor.GraphModel


setEndVertex(Vertex) - Method in class org.bioalg.graphprocessor.BranchModel


setFixed(boolean) - Method in class org.bioalg.graphprocessor.Vertex


setNodes(ArrayList<Vertex>) - Method in class org.bioalg.graphprocessor.Path


setP1(Point) - Method in class org.bioalg.graphprocessor.Edge


setP2(Point) - Method in class org.bioalg.graphprocessor.Edge


setStart2endPath(ArrayList<Path>) - Method in class org.bioalg.graphprocessor.BranchModel


setStartVertex(Vertex) - Method in class org.bioalg.graphprocessor.BranchModel


setType(Edge.EDGE\_TYPE) - Method in class org.bioalg.graphprocessor.Edge


setType(Vertex.VERTEX\_TYPE) - Method in class org.bioalg.graphprocessor.Vertex


setVertex1(Vertex) - Method in class org.bioalg.graphprocessor.EdgeModel


setVertex2(Vertex) - Method in class org.bioalg.graphprocessor.EdgeModel


setVertices(ArrayList<Vertex>) - Method in class org.bioalg.graphprocessor.GraphModel


showError(GraphModel.GraphErrors) - Method in class org.bioalg.graphprocessor.GraphModel


size() - Method in class org.bioalg.graphprocessor.Path


## T

toGraphModel() - Method in class org.bioalg.graphprocessor.Graph
:   Convert this graph to a graph model.

toString() - Method in class org.bioalg.graphprocessor.EdgeModel


toString() - Method in class org.bioalg.graphprocessor.GraphModel


toString() - Method in class org.bioalg.graphprocessor.Vertex


## V

valueOf(String) - Static method in enum org.bioalg.graphprocessor.Edge.EDGE\_TYPE
:   Returns the enum constant of this type with the specified name.

valueOf(String) - Static method in enum org.bioalg.graphprocessor.GraphModel.GraphErrors
:   Returns the enum constant of this type with the specified name.

valueOf(String) - Static method in enum org.bioalg.graphprocessor.Vertex.VERTEX\_TYPE
:   Returns the enum constant of this type with the specified name.

values() - Static method in enum org.bioalg.graphprocessor.Edge.EDGE\_TYPE
:   Returns an array containing the constants of this enum type, in
    the order they are declared.

values() - Static method in enum org.bioalg.graphprocessor.GraphModel.GraphErrors
:   Returns an array containing the constants of this enum type, in
    the order they are declared.

values() - Static method in enum org.bioalg.graphprocessor.Vertex.VERTEX\_TYPE
:   Returns an array containing the constants of this enum type, in
    the order they are declared.

verfiyModel() - Method in class org.bioalg.graphprocessor.GraphModel


Vertex - Class in org.bioalg.graphprocessor
:   A graph vertex is a point with a given type.

Vertex() - Constructor for class org.bioalg.graphprocessor.Vertex


Vertex(int, int, Vertex.VERTEX\_TYPE) - Constructor for class org.bioalg.graphprocessor.Vertex


Vertex(Point, Vertex.VERTEX\_TYPE) - Constructor for class org.bioalg.graphprocessor.Vertex


Vertex(Element) - Constructor for class org.bioalg.graphprocessor.Vertex


Vertex.VERTEX\_TYPE - Enum in org.bioalg.graphprocessor
:   The types of the vertices.

A B C E F G H I M O P R S T V

- Package
- Class
- Tree
- Deprecated
- Index
- Help

*org.bioalg.graphprocessor 1.0*

- Prev
- Next

- Frames
- No Frames

- All Classes
